# Supplementary material for: Structure-Based Design of an RNase Chimera for Antimicrobial Therapy
Source: Int J Mol Sci. 2021 Dec 22;23(1):95. doi: 10.3390/ijms23010095 (PMC8745102; doi:10.3390/ijms23010095)
Supplement: Supplementary file 1 [file ijms-23-00095-s001.zip › ijms-1492708-supplementary/RNase_31_Supplemental_final.pdf]

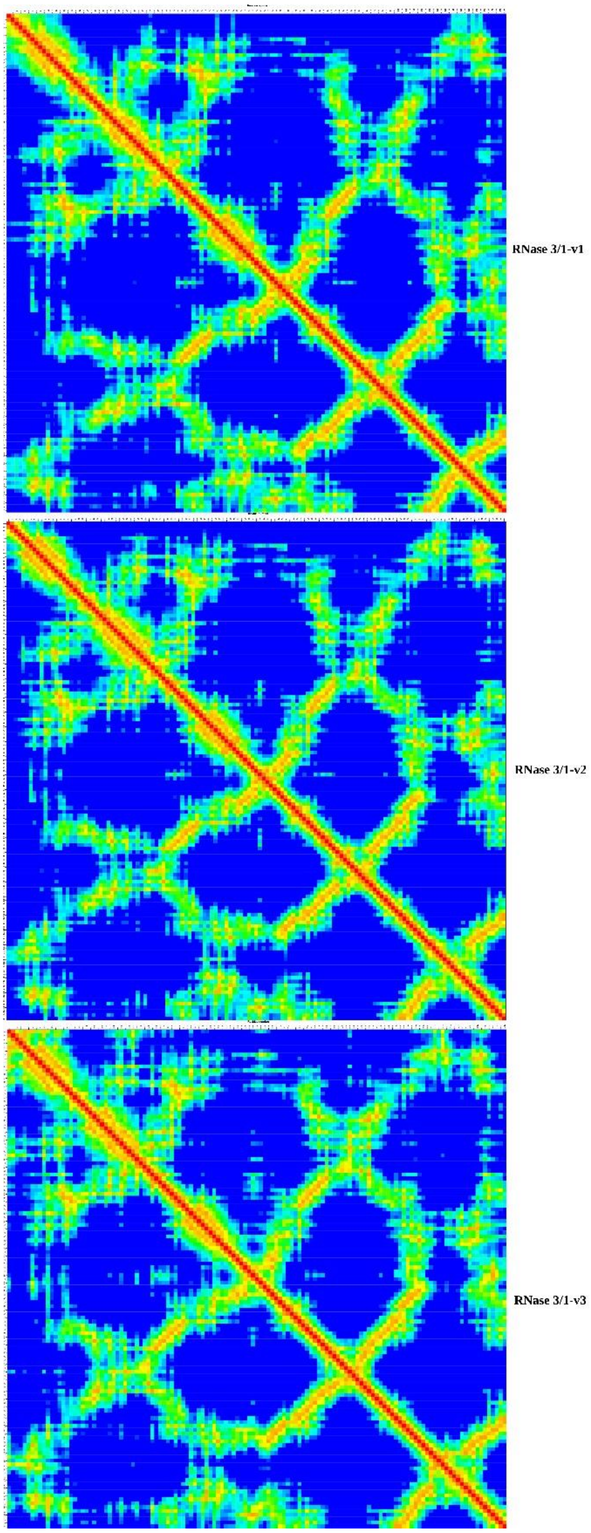

Figure S1. Whole trajectory averaged inter-residual distance maps for RNase 3/1 versions. From left to right, RNase 3/1-v1, RNase 3/1-v2 and RNase 3/1-v3 distance maps. Residue numbering, secondary structure labels and active site residues (red arrow) are shown at the bottom of each panel. See also the additional supplementary maps for each variant at high-resolution.

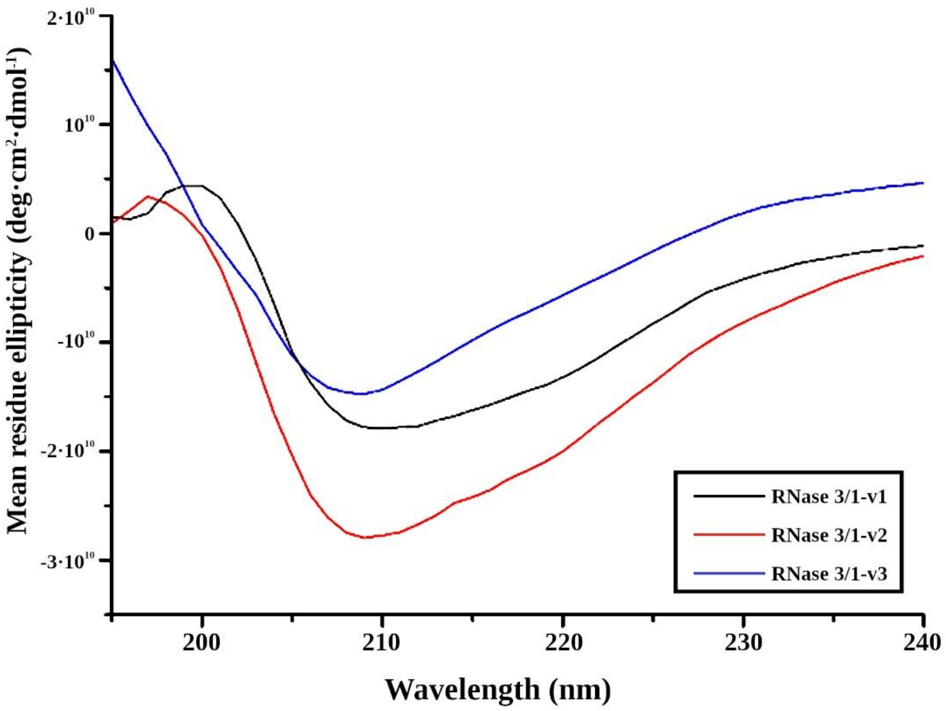

	RNase 3/1-v1	RNase 3/1-v2	RNase 3/1-v3
α-helix	18.3	14.1	18.6
β-sheet	59.4	41.6	62.8
Random coil	16.6	28.7	18.6

Figure S2. Circular dichroism spectra of the three versions of RNase 3/1. The embedded table indicates the estimated % of secondary structures for each construct.

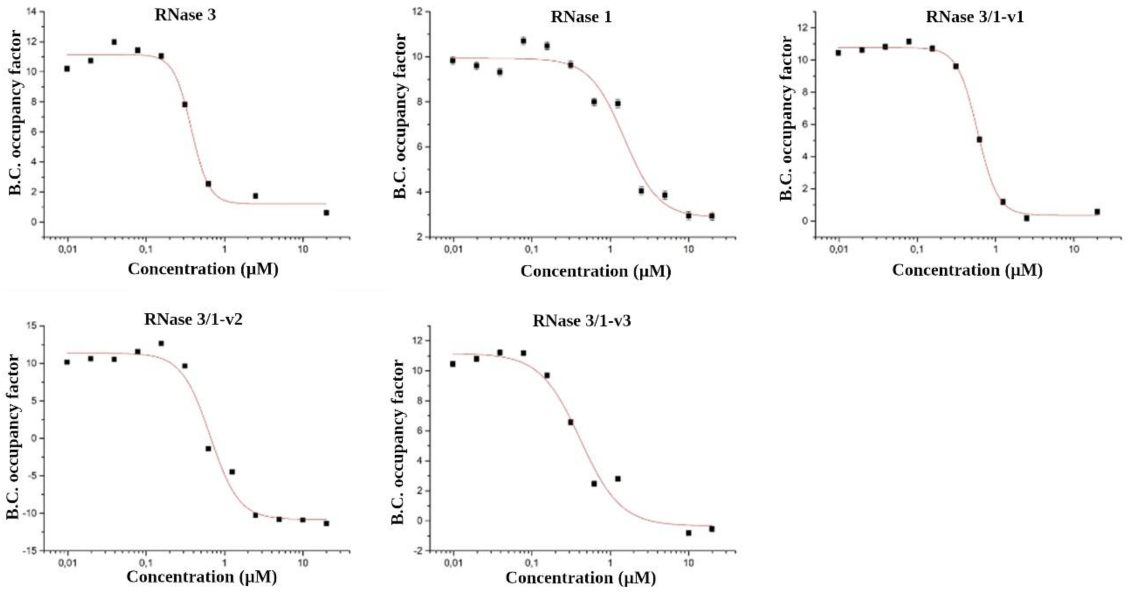

Figure S3. LPS binding activity of RNases 1, 3 and the three versions of RNase 3/1 after 1 h of incubation. Dose-response curves were calculated using *OriginPro 8 statistical software*. Results are the average of three replicates.

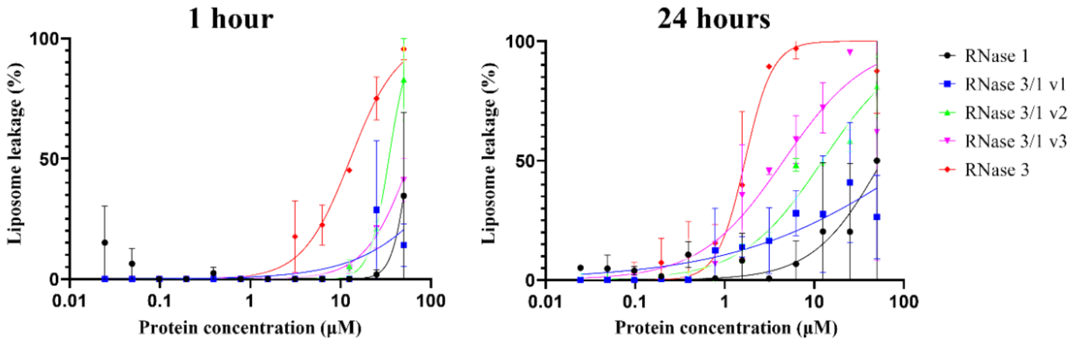

Figure S4. Liposome leakage activity of RNases 1, 3 and the three versions of RNase 3/1 after 1 and 24 hours of exposure. Dose-response curves were calculated using *GraphPad Prism 9*. Error bars show the standard error of the mean (SEM).

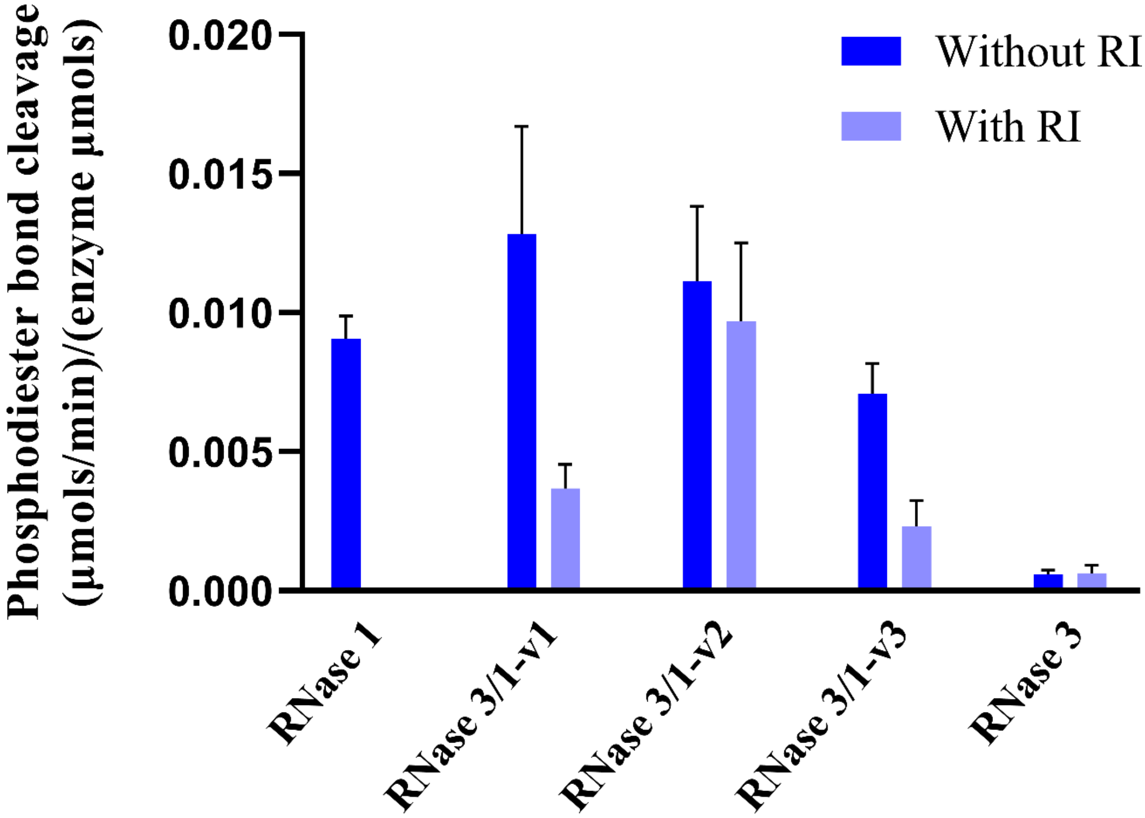

Figure S5. Ribonuclease inhibitor (RI) percentage of inhibition was determined by comparing the catalytic activity against CpA with or without presence of the inhibitor. Values represent the activity in µmols of product per µmol of enzyme. Results are shown from at least 3 replicates (mean ± SEM).

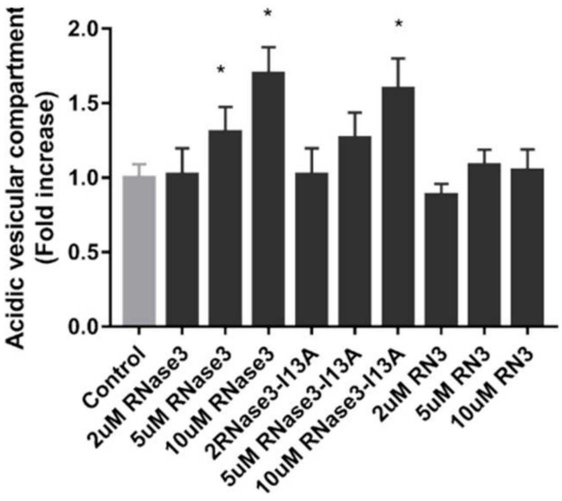

Figure S6. Autolysosome formation measured by AO staining. Results are shown from 8 independent experiments (mean ± SD).

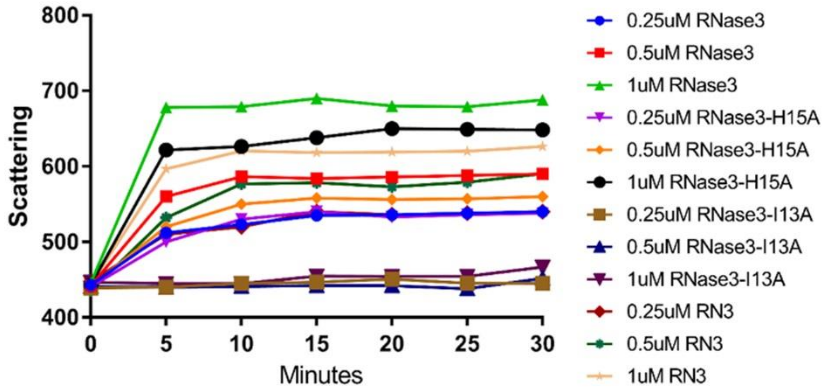

Figure S7. Liposome aggregation by different timing points and/or concentration of protein or peptide. Comparison of kinetic aggregation profile of liposomes by several concentrations of RNase 3 wild-type, mutants and RN3 peptide protein (from 0.25 μM to 1 μM). Protein or peptide was incubated with 200 μM of liposomes at room temperature, the scattering intensity was recorded every 5 minutes, while no protein added is indicated as time 0.

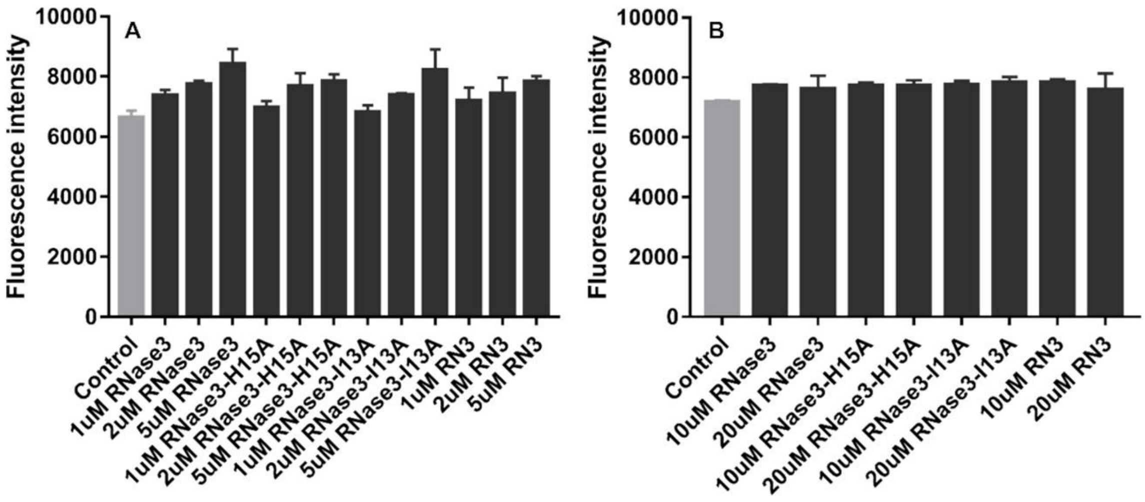

Figure S8. RAW 264.7 macrophages were infected with *M. aurum* and treated with **(A)** 1-5 µM and **(B)** 10 µM of proteins (RNase 3, I13A, H15A and W35A) and the RN3(1-45) peptide for 1 hour and then stained for 15 minutes by Thioflavin-S.

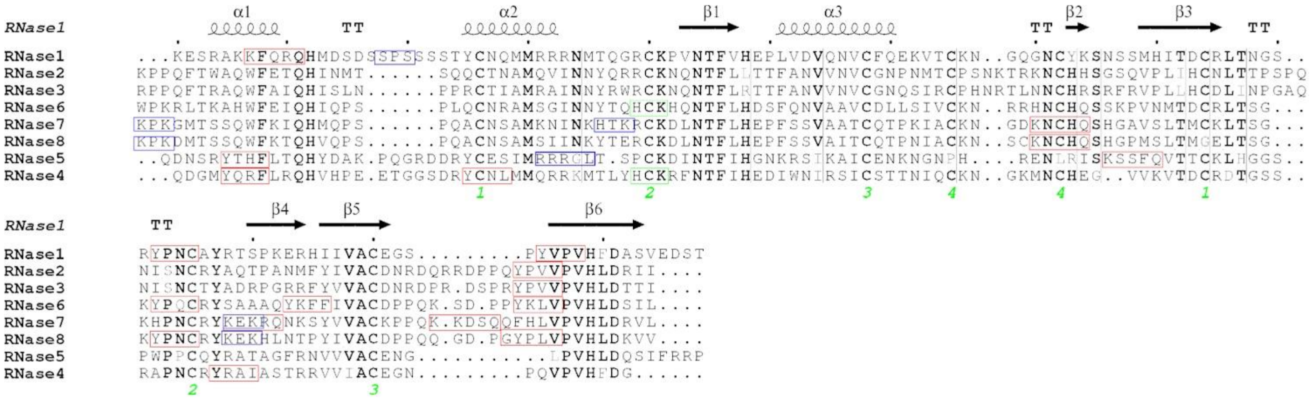

Figure S9. Putative signal tag of the subcellular localization of human RNases. Red box indicates the tag for lysosome; blue for endoplasmic reticulum and green for nucleus. Green numbers indicate disulphide bonds. The tags were identified by *LocSigDB* (Negi et al., 2015). Sequences are detailed in table S1.

Table S1. Prediction of the subcellular localization of human RNases by signal tag

	Signal	Aminoacid Number/Tag	Localization
**RNase 1**	Yx{2}[VILFWCM]	91-95/YPNC;<br>114-118/YVPV	Lysosome
	Kx{3}Q	6-11/KFQRQ	Lysosome
	SPS	17-20/SPS	Nucleus
**RNase 2**	Yx{2}[VILFWCM]	122-126/YPVV	Lysosome
**RNase 3**	Yx{2}[VILFWCM]	121-125YPVV	Lysosome
**RNase 4**	Yx{2}[VILFWCM]	4-8/YQRF;<br>23-27/YCNL;<br>93-97/YRAT	Lysosome
	[HK]x{1}K	37-40/HCK	Endoplasmic reticulum
**RNase 5**	Yx{2}[VILFWCM]	5-9/YTFH	Lysosome
	Kx{3}Q	72-77/KSSFQ	Lysosome
	RRRGL	30-35/RRRGL	Nucleus
**RNase 6**	Yx{2}[VILFWCM]	87-91/YPQC;<br>98-102/YKFF;<br>115-119/YKLV	Lysosome
	[HK]x{1}K	35-38/HCK	Endoplasmic reticulum
**RNase 7**	Kx{3}Q	66-71/KNCHQ;<br>93-98/KEKRQ;<br>110-115/KKDSQ	Lysosome
	[HK]x{1}K	0-3/KPK;<br>32-35/HTK;<br>93-96/KEK	Endoplasmic reticulum
**RNase 8**	Yx{2}[VILFWCM]	87-91/YPNC;<br>115-119/YPLV	Lysosome
	Kx{3}Q	66-71/KNCHQ	Lysosome
	GYx{2}[VILFWCM]	114-119/GYPLV	Lysosome

[HK]x{1}K	0-3/KPK;<br>93-96/KEK	Endoplasmic reticulum
