# Supplementary figures and images for: Structure-Based Design of an RNase Chimera for Antimicrobial Therapy
Source: Int J Mol Sci. 2021 Dec 22;23(1):95. doi: 10.3390/ijms23010095 (PMC8745102; doi:10.3390/ijms23010095)

RNase 3/1-v1 distance map

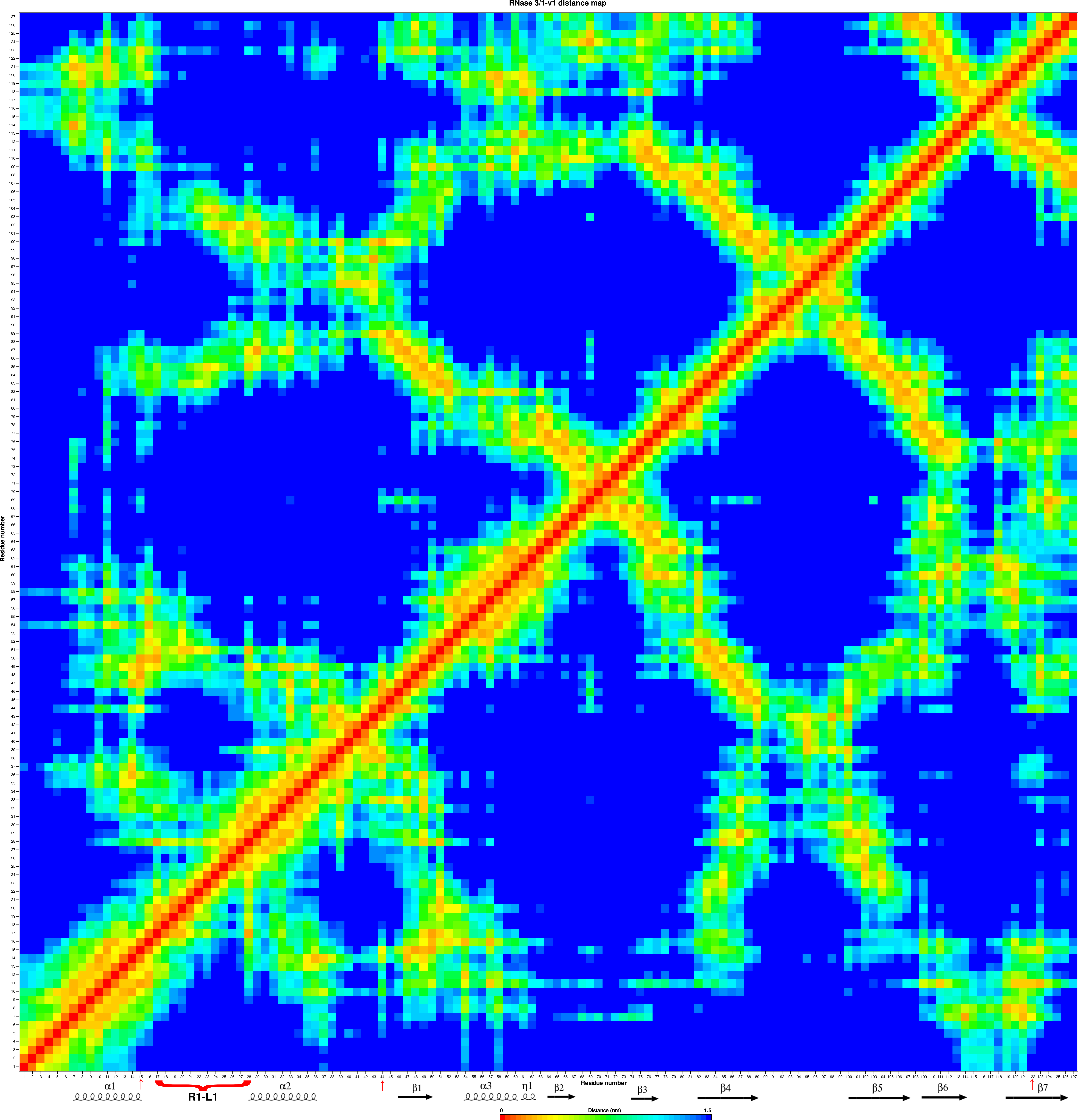

Supplement: Supplementary file 1 [file ijms-23-00095-s001.zip › ijms-1492708-supplementary/supplementary files/R31_v1_distance_map.pdf]

RNase 3/1-v2 distance map

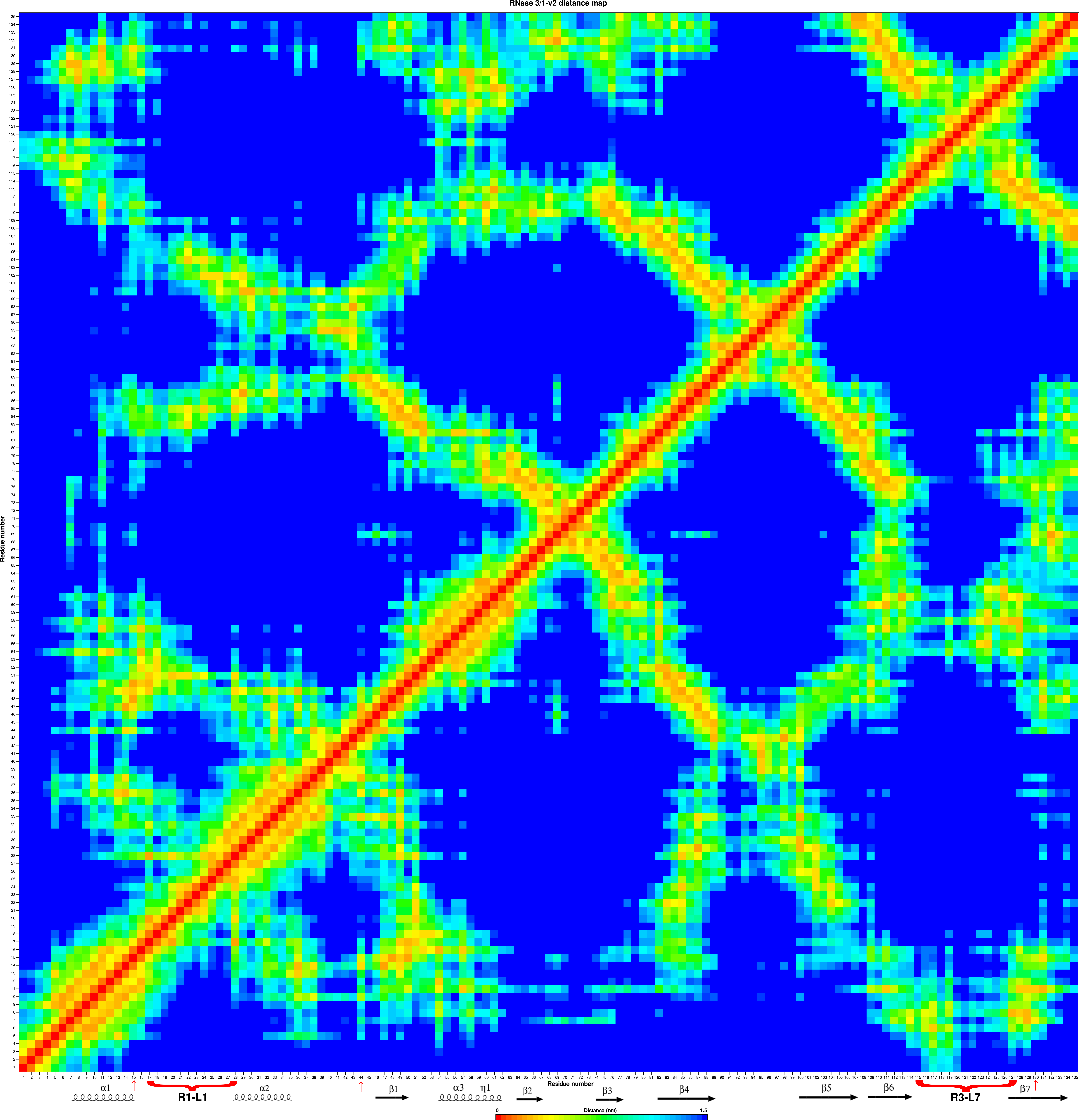

Supplement: Supplementary file 1 [file ijms-23-00095-s001.zip › ijms-1492708-supplementary/supplementary files/R31_v2_distance_map.pdf]

RNase 3/1-v3 distance map

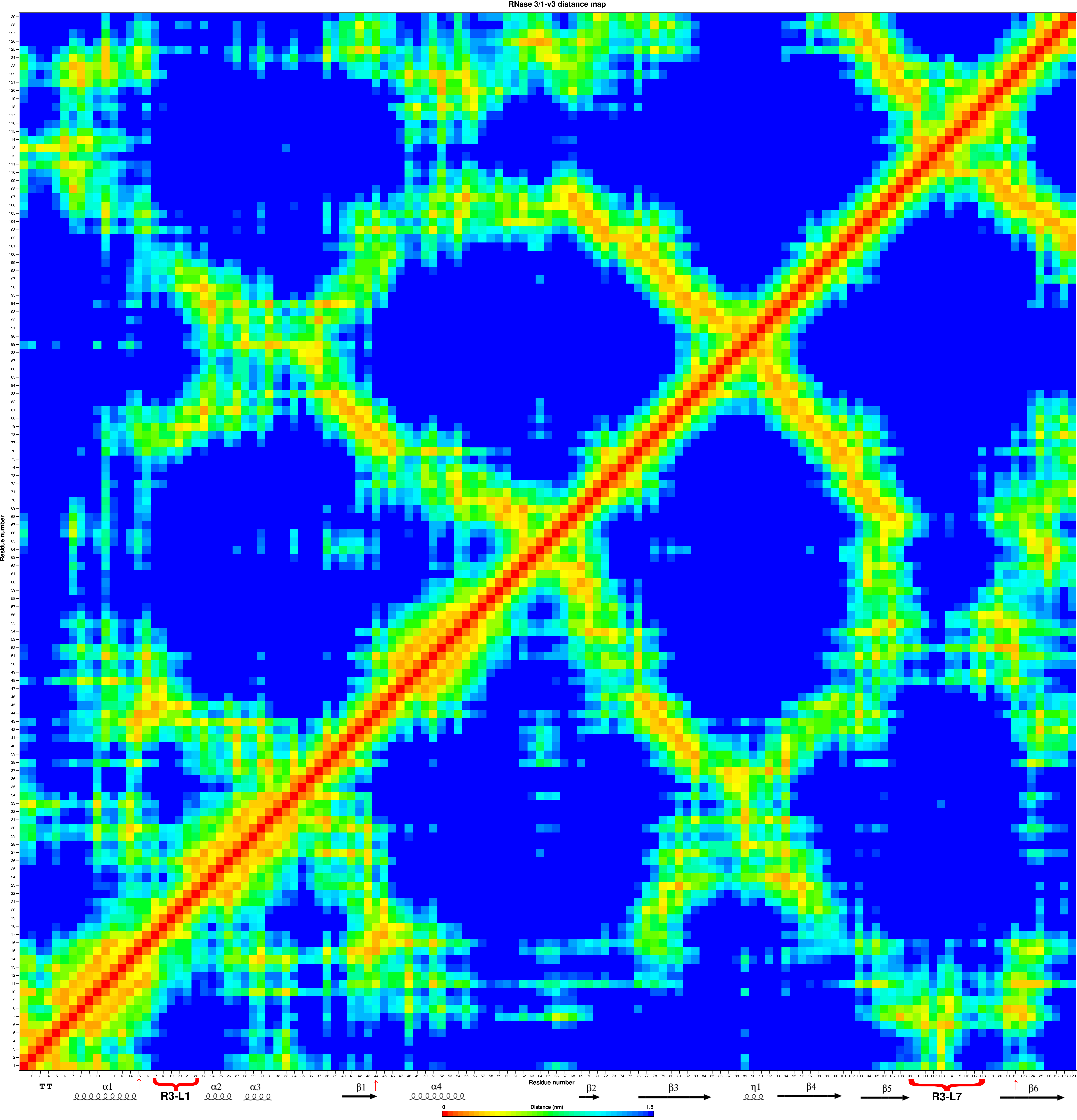

Supplement: Supplementary file 1 [file ijms-23-00095-s001.zip › ijms-1492708-supplementary/supplementary files/R31_v3_distance_map.pdf]
